# Comparison of food consumption and nutrient intake assessed with three dietary assessment methods: results of the German National Nutrition Survey II

**DOI:** 10.1007/s00394-017-1583-z

**Published:** 2017-11-30

**Authors:** Andrea Straßburg, Marianne Eisinger-Watzl, Carolin Krems, Alexander Roth, Ingrid Hoffmann

**Affiliations:** 1 Department of Nutritional Behaviour, Max Rubner-Institut, Federal Research Institute of Nutrition and Food, Haid-und-Neu-Str. 9, 76131 Karlsruhe, Germany; 2 Department of Physiology and Biochemistry of Nutrition, Max Rubner-Institut, Federal Research Institute of Nutrition and Food, Haid-und-Neu-Str. 9, 76131 Karlsruhe, Germany; 3 Present Address: Centre of Child and Adolescent Psychiatry of the University of Zurich, Neumünsterallee 9, 8032 Zurich, Switzerland

**Keywords:** Diet history interviews, 24-h recalls, Weighed food records, Food consumption, Nutrient intake, Underreporting

## Abstract

**Purpose:**

Comparison of food consumption, nutrient intake and underreporting of diet history interviews, 24-h recalls and weighed food records to gain further insight into specific strength and limitations of each method and to support the choice of the adequate dietary assessment method.

**Methods:**

For 677 participants (14–80 years) of the German National Nutrition Survey II confidence intervals for food consumption and nutrient intake were calculated on basis of bootstrapping samples, Cohen’s *d* for the relevance of differences, and intraclass correlation coefficients for the degree of agreement of dietary assessment methods. Low energy reporters were identified with Goldberg cut-offs.

**Results:**

In 7 of 18 food groups diet history interviews showed higher consumption means than 24-h recalls and weighed food records. Especially mean values of food groups perceived as socially desirable, such as fruit and vegetables, were highest for diet history interviews. For “raw” and “cooked vegetables”, the diet history interviews showed a mean consumption of 144 and 109 g/day in comparison with 68 and 70 g/day in 24-h recalls and 76 and 75 g/day in weighed food records, respectively. For “fruit”, diet history interviews showed a mean consumption of 256 g/day in comparison with 164 g/day in 24-h recalls and 147 g/day in weighed food records. No major differences regarding underreporting of energy intake were found between dietary assessment methods.

**Conclusions:**

With regard to estimating food consumption and nutrient intake, 24-h recalls and weighed food records showed smaller differences and better agreement than pairwise comparisons with diet history interviews.

## Introduction

Each dietary assessment method has its own strengths and limitations. Depending on these, the method which suits best for a special research focus has to be chosen [1–3]. The more is known about strengths and limitations, the better the choice of the dietary assessment method can be made. Furthermore, for interpretation and comparison of existing studies and nutrition surveys, methodological aspects need to be considered.

In Europe, most countries conduct national food consumption surveys. For several years, there have been efforts to harmonize the assessment of food consumption in Europe to allow international comparisons [4–7]. 24-h recalls and food records are currently most often used in population-based dietary surveys in Europe [7–9] and were also applied in the German National Nutrition Survey (NVS) II. Because of decreasing response rates in national surveys, the burden for the participants should be kept at a minimum. Therefore, the current EFSA guideline for a pan-European dietary survey (EU Menu) states that food consumption information should be collected for two non-consecutive days by 24-h recalls for adults [7]. In addition, the diet history interview was applied in NVS II. A comparison of food consumption data of diet history interviews and 24-h recalls of the NVS II was recently published [10]. The present paper extends the comparison to all three applied methods in a subgroup of 677 participants also considering energy and nutrient intake as well as underreporting. Possible reasons for differences or agreement in food consumption and nutrient intake results will be discussed to give further insight in special strengths and limitations of each dietary assessment method and to support the choice of the adequate dietary assessment method.

## Materials and methods

### Study design

The German Federal Ministry of Food, Agriculture and Consumer Protection commissioned the Max Rubner-Institut to conduct the National Nutrition Survey II which was realised from November 2005 to January 2007. The survey is representative for the German-speaking population 14–80 years of age living in private households. A two-stage random sampling procedure was applied. The response rate was 42%. The study design is described in detail elsewhere [11]. Within the NVS II, food consumption was assessed using three dietary assessment methods: diet history interviews (*n* = 15.371), 24-h recalls (*n* = 13.926), and weighed food records (*n* = 975). Participants who completed all three dietary assessment methods were included in the present study (*n* = 677).

### Dietary assessment methods

#### Diet history interviews

At study centres, usual food consumption of 15,371 participants was assessed with diet history interviews. Specially trained interviewers (mostly nutritionists) used the software program DISHES (Diet Interview Software for Health Examination Studies) developed by the Robert Koch-Institut, Berlin, for the German Nutrition Survey 1998 [12]. Small modifications to the software due to requirements of the NVS II were made [13], e.g., regarding a non-user list. The open-ended interview follows the daily meal structure and covers usual food consumption of the past 4 weeks. Food items were directly linked with the German Nutrient Database (BLS). Quantification of portion sizes was accomplished with household measurements, models of tableware (cups, glasses, spoons, plates, and bowls), and a 30 page picture book with different portion sizes of food items. The picture book is an excerpt of the original EPIC-SOFT^1^ picture book [14] modified for the NVS II, e.g., new weights for the shapes of bread were included. To increase data quality, a plausibility check to identify and correct for input errors was conducted. Several times during the survey quality assurance checks were made by external supervisors [11].

#### Weighed food records

In each sample point, 4–5 participants were randomly chosen to conduct weighed food records. The aim was to achieve 1000 weighed food records. 1021 participants returned their food records of which 46 were incomplete. As a result, 975 participants accomplished two weighed food records, each on four consecutive days (including weekends). During the visit at the study centres, participants were instructed by the trained interviewers. They received standardized booklets for recording and a digital kitchen scale to weigh portion sizes of consumed foods at home (Soehnle venezia, max. 2000 g at 1 g precision providing a tare function). Participants were asked to estimate portion size when weighing was not possible. The quantities of about 25% of the recorded food items were estimated. Completed food records were mailed back. The chosen setting of two times 4 days of recording caused an overlap of Wednesdays and Saturdays. It is known that food consumption differs between weekdays and weekends [12, 15, 16]. Internal analyses showed that intakes of energy and carbohydrates on Saturdays were significantly higher than the intakes on weekdays (data not shown). Therefore, food consumption of each day of the week was weighted to achieve a homogenous distribution of week days. Weighed food records started within a mean of 7 days after the visit at the study centre and were finished within a mean of 22 days.

#### 24-h recalls

For the 24-h recalls, participants were asked in a telephone interview about their food and beverage consumption of the previous day. In total, 13,926 participants finished two 24-h recalls. Trained interviewers of a specialized call centre used the software program EPIC-SOFT, which was developed for the European Prospective Investigation into Cancer and Nutrition by the International Agency for Research on Cancer (IARC) [14]. Corresponding to a first so-called quick list of the consumed foods in chronological order, the software program supports specification of the reported food items in several steps. Quantification of portion sizes was carried out with the EPIC-SOFT picture book (identical with the one used for the diet history interview), household measurements as well as standard units. EPIC-SOFT includes control questions and integrated quality checks [14, 17, 18]. The randomly sampled assessment days covered weekdays and weekend-days with 75 and 25%, respectively. The first 24-h recall was conducted on average 9 days after the participants finished the weighed food records, the second 24-h recall on average 14 days later. Altogether, the average time span for completing all three dietary assessment methods was 45 days.

### Assessment of nutrient intake

To calculate energy and nutrient intakes the German nutrient database (BLS), version 3.02 was used.

### Assessment of under- and overreporting

To study the extent of under- and overreporting, the proportions of low- and high-energy reporting were assessed using the cut-off points described by Goldberg et al. [19] adapted by Black [20]. Estimation of under- and overreporting is based on the ratio of reported energy intake and calculated resting metabolic rate. Resting metabolic rate was determined by the formula of Müller et al. [21] including sex, age, body height, and weight for adolescents 14–17 years of age and sex, age and body weight for adults. Body height and weight were measured at study centres. To define the proportions of low- and high-energy reporting for each assessment method, the cut-off 2 [19], which considers sample size and number of assessment days, was calculated for each method. The calculated cut-off points for underreporting were 1.09 for diet history interviews, 0.97 for 24-h recalls, and 1.06 for weighed food records. Cut-off points for overreporting were 2.21 for diet history interviews, 2.49 for 24-h recalls, and 2.27 for weighed food records.

### Standardisation procedures

Within both retrospective methods (diet history interviews and 24-h recalls), equal estimation of serving sizes was supported by applying the identical picture book. However, the standard portions were sometimes unequal due to different software embedded values. Because of numerous possibilities how foods are eaten (e.g., an apple or apple as an ingredient of an apple cake) and how portion sizes could be quantified (e.g., household measurements, standard units, models of tableware, and the picture book) those differently embedded values for the standard portions only have a minor influence on the present study.

Diet history interviews, 24-h recalls and weighed food records differ in their procedures to capture recipes. To achieve a consistent food group categorization (Appendix), the level of recipe aggregation of 24-h recalls served as the standard. About 1200 recipes (45%) of the diet history interviews were disaggregated (e.g., lasagne) and about 1700 recipes (61%) of the weighed food records were aggregated (e.g., cakes or dressings) corresponding to the interviewee statements. Diet history interviews and weighed food records are both assumed to mirror habitual consumption, also covering rarely eaten foods. In contrast, the 24-h recalls measure short-term consumption. To estimate the distribution of usual food consumption on the basis of two 24-h recalls, the Multiple Source Method (MSM) was applied [22, 23].

### Data analysis

**Fig. 1:**
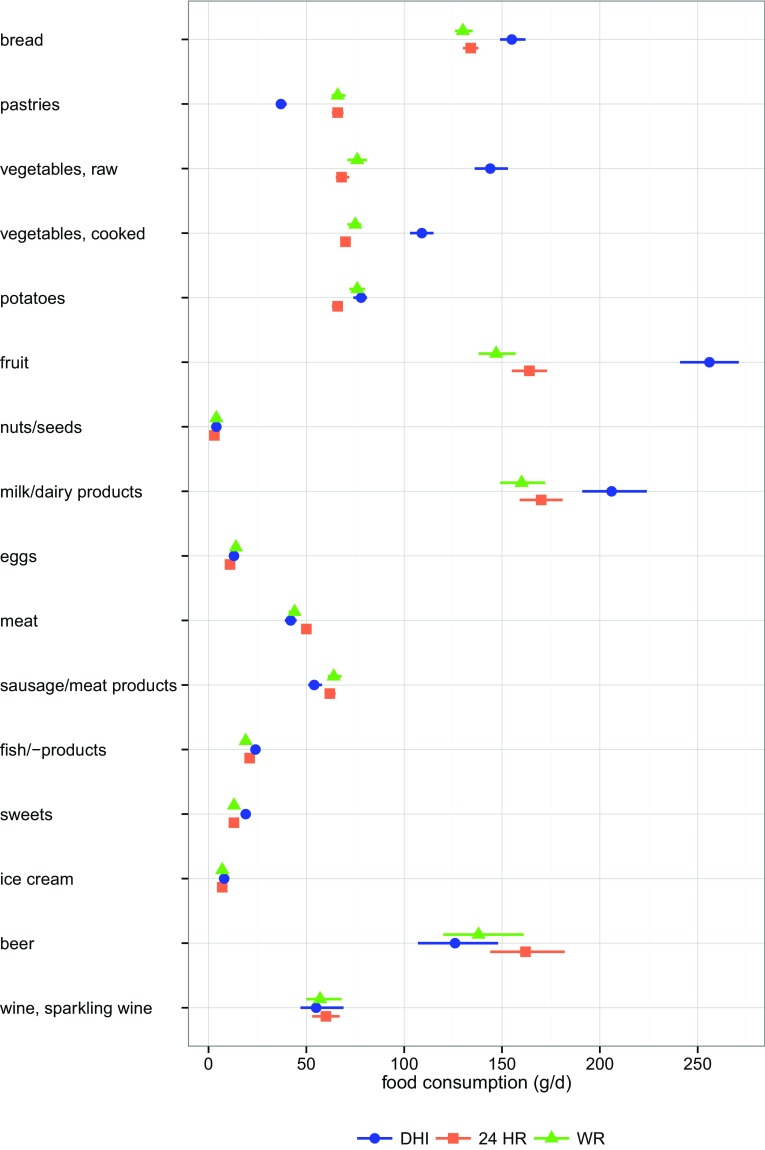
Food consumption (mean, 95% confidence intervals) of the three dietary assessment methods

**Fig. 2:**
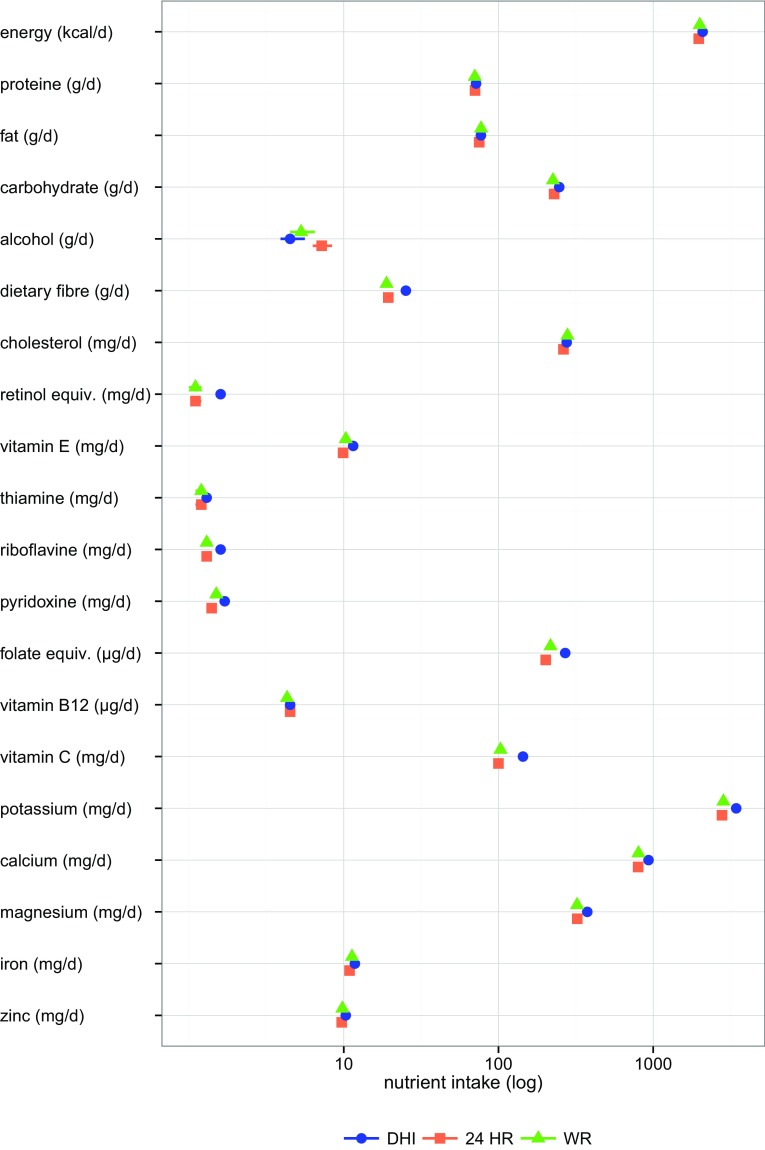
Nutrient intake (median, 95% confidence intervals) of the three dietary assessment methods

Neither food consumption nor nutrient intake estimates were normally distributed. Results of food consumption and nutrient intake are presented as arithmetic mean and median. In addition, in Fig. 1, 95% confidence intervals (CI) for the mean and, in Fig. 2, 95% CI for the median are shown. For interpretation of food consumption data, the mean is used, because food groups consumed by less than 50% of the participants lead to medians with value ‘0’. Pairwise differences between two methods are presented as means with corresponding 95% CI. Data could not be normalized by log-transformation, so for calculating confidence intervals, the bootstrapping procedure was used. Bootstrapping is a distribution-independent resampling method [24] of which bias controlled results were taken. Cohen’s *d* was calculated for equal sample sizes to determine the relevance of the obtained differences. The higher the value in the range from 0 to 1 the stronger is the assumed effect size. Differences between underreporters and plausible reporters within one assessment method were compared by CI and the Mann–Whitney *U* test.

The intraclass correlation coefficient (ICC, two-way mixed) was used to describe the degree of agreement between the individual food consumption estimates measured by the dietary assessment methods [25, 26]. The ICC ranges were defined as follows: ≤ 0.20 weak, 0.21–0.40 fair, 0.41–0.60 moderate, 0.61–0.80 strong, and 0.81–1.00 almost perfect. For the interpretation of the ICC, it has to be considered that 24-h recall values estimated with MSM do not show the real distribution of usual consumption. Instead, the calculated values are rather a theoretical assumption for a possible distribution of usual consumption. For ICC calculation, PASW Statistics Version 17.0 (SPSS Inc., Chicago, IL, USA) was used. The other statistical analyses were performed using SAS version 9.2 (SAS Institute, Inc., Cary, NC; USA). Differences are considered to be significant at a level of *P* < 0.05. Regarding confidence intervals, differences are considered to be significant if they do not overlap or for the calculated differences between two dietary assessment methods if confidence intervals include zero.

## Results

### Sample characteristics

In the present study, the percentage of women and the level of education are higher, while the percentage of smokers is lower compared to the total study sample of NVS II (Table 1).

**Table 1:** Characteristics of the presented subsample and all participants of NVS II

	Subsample for comparison (*n* = 677)	NVS II (*n* = 19,329)
Sex		
Men (%)	41.1	46.2
Women (%)	58.9	53.8
Age (years)		
Mean (SE)	47.7 (0.65)	45.8 (0.13)
Body mass index (kg/m^2^)		
Mean (SE)	26.2 (0.19)^a^	26.1 (0.04)^b^
Marital status		
Married (%)	64.1	59.5
Education		
≥ 10 years (%)	61.3	58.4
Smoking		
Smokers (%)	18.5	26.9

*SE* standard error

^a^*n* = 663

^b^*n* = 14,586

### Comparison on the level of food consumption

#### Mean values

For the diet history interviews, seven higher (bread, raw and cooked vegetables, fruit, milk/dairy products, sweets and water) and two lower (pastries, sausage/meat products) consumption means were found compared to the results of weighed food records and 24-h recalls (Fig. 1). Lowest coffee/tea consumption was assessed with the weighed food records.

#### Differences

Overall, differences between diet history interviews and 24-h recalls are largest, whereas differences between 24-h recalls and weighed food records are lowest (Table 2). All three pairwise comparisons showed significant differences (confidence intervals for differences do not include zero) for the food groups raw and cooked vegetables, fruit, milk/dairy products, and fish/-products. According to Cohen’s *d*, the relevance of the differences is highest for the food groups pastries, raw and cooked vegetables, and fruit for pairwise differences with the diet history interviews as well as for the difference between 24-h recalls and weighed food records for coffee/tea.

**Table 2:** Food consumption and pairwise differences between the dietary assessment methods with effect size estimates

	DHI		24HR		WR		Difference of means 24HR-DHI				Difference of means DHI-WR				Difference of means 24HR-WR			
	Mean	Median	Mean	Median	Mean	Median	Mean	CI	%^a^	Effect	Mean	CI	%^b^	Effect	Mean	CI	%^c^	Effect
	(g/day)	(g/day)	(g/day)	(g/day)	(g/day)	(g/day)	(g/day)	(g/day)		size^d^	(g/day)	(g/day)		size^d^	(g/day)	(g/day)		size^d^
Bread	155	140	134	126	130	122	− 21.6	− 27.8; − 15.9	− 13.9	0.3	25.0	19.8; 30.8	19.2	0.3	3.4	− 0.7; 7.4	2.5	0.1
Pastries	37	29	66	64	66	56	28.8	25.4; 31.9	77.2	0.7	− 29.0	− 32.8; − 25.4	− 43.8	0.6	− 0.3	− 4.2; 3.5	− 0.4	0.0
Vegetables, raw	144	120	68	59	76	59	− 75.9	− 84.0; − 68.4	− 52.7	0.7	68.5	61.4; 75.9	90.7	0.7	− 7.4	− 11.8; − 3.2	− 10.8	0.1
Vegetables, cooked	109	90	70	66	75	65	− 39.0	− 45.3; − 33.5	− 35.9	0.5	34.1	28.2; 40.3	45.7	0.4	− 4.9	− 8.7; − 1.4	− 7.0	0.1
Potatoes	78	70	66	60	76	67	− 12.1	− 15.9; − 8.6	− 15.6	0.2	1.4	− 2.4; 5.5	1.9	0.0	− 10.7	− 14.5; − 6.8	− 16.3	0.2
Fruit	256	223	164	146	147	117	− 92.2	− 106.1; − 79.6	− 36.1	0.5	108.9	96.8; 122.5	74.1	0.6	16.7	8.0; 24.8	10.2	0.2
Nuts/seeds	4	0	3	0	4	0	− 0.9	− 1.9; − 0.1	− 22.0	0.1	0.6	− 0.1; 1.6	18.4	0.1	− 0.3	− 0.9; 0.4	− 8.2	0.0
Milk/dairy products	206	152	170	135	160	128	− 36.5	− 50.9; − 23.5	− 17.7	0.2	46.3	33.5; 60.1	29.0	0.3	9.8	0.6; 19.0	5.8	0.1
Eggs	13	10	11	6	14	10	− 2.0	− 3.4; − 0.9	− 15.2	0.1	− 0.3	− 1.4; 0.9	− 2.4	0.0	− 2.3	− 3.5; − 1.2	− 20.9	0.1
Meat	42	34	50	46	44	39	8.4	5.3; 11.1	20.2	0.2	− 2.0	− 4.9; 1.3	− 4.5	0.0	6.4	3.7; 9.1	12.9	0.2
Sausages/meat products	54	44	62	58	64	55	8.1	4.9; 11.3	14.9	0.2	− 9.9	− 13.1; − 6.6	− 15.4	0.2	− 1.8	− 5.0; 1.5	− 2.8	0.0
Fish/-products	24	19	21	20	19	10	− 2.7	− 4.5; − 1.1	− 11.6	0.1	5.1	3.2; 6.9	27.5	0.2	2.3	0.5; 4.0	11.3	0.1
Sweets	19	11	13	5	13	7	− 6.0	− 8.2; − 4.2	− 31.7	0.2	6.1	4.4; 8.2	47.7	0.2	0.1	− 1.4; 1.5	0.9	0.0
Ice cream	8	2	7	2	7	0	− 1.2	− 2.7; 0.2	− 14.8	0.1	1.0	− 0.4; 2.5	14.4	0.1	− 0.2	− 1.5; 1.1	− 2.4	0.0
Water	1226	1100	1003	935	967	881	− 223.0	− 276.0; − 170.5	− 18.2	0.3	258.9	205.3; 313.6	26.8	0.4	35.9	− 6.8; 76.8	3.6	0.1
Coffee/tea	510	450	516	511	404	383	6.1	− 20.1; 30.8	1.2	0.0	105.6	81.2; 131.5	26.1	0.3	111.7	92.6; 129.8	21.7	0.5
Beer	126	12	162	55	138	0	36.6	20.8; 51.8	29.1	0.2	− 12.4	− 26.7; 2.1	− 9.1	0.1	23.9	9.3; 38.1	14.7	0.1
Wine, sparkling wine	55	13	60	23	57	0	5.2	− 5.3; 12.2	9.4	0.0	− 2.5	− 8.9; 7.1	− 4.3	0.0	2.7	− 5.6; 9.2	4.5	0.0

DHI diet history interviews, 24HR 24-h recalls, WR weighed food records, CI confidence intervals calculated with bootstrap method

^a^DHI = 100%

^b^WR = 100%

^c^24HR = 100%

^d^Cohen’s *d*

Food consumption data comprise a substantial portion of zero values from non-consumption of food items (Table 3). Rarely or seasonal consumed food groups (e.g., nuts/seeds, ice cream) and alcoholic beverages exhibit the highest percentages of zero consumption in all three dietary assessment methods. All in all, the highest proportions of non-consumption in the present study are seen for weighed food records. It has to be considered that the estimation of the usual food consumption distribution with MSM for 24-h recalls led to lower proportions of zero values in comparison with the original data.

**Table 3:** Proportion of non-consumption for each dietary assessment method

	Diet history interviews (%)	24-h recalls (%)	weighed food records (%)
Bread	0	0	0
Pastries	9	5	7
Vegetables, raw	1	1	4
Vegetables, cooked	0	0	1
Potatoes	3	2	4
Fruit	3	2	6
Nuts/seeds	62	55	62
Milk/dairy products	0	0	4
Eggs	10	8	18
Meat	4	3	8
Sausages/meat products	4	3	3
Fish/-products	14	13	33
Sweets	19	14	34
Ice cream	46	43	66
Water	0	3	6
Coffee/tea	12	10	11
Beer	48	45	55
Wine, sparkling wine	24	35	51

#### Agreement: ICC

Strong agreement between all three dietary assessment methods was reached for coffee/tea and beer (Table 4). Moderate agreement was found for (sparkling) wine, water, milk/dairy products, sausages/meat products, bread, and fruit. Weak agreement was seen for the food group cooked vegetables, while the other food groups reached a fair agreement. The paired analysis showed the strongest correlation coefficients between 24-h recalls and weighed food records in comparison with both other combinations with diet history interviews.

**Table 4:** Intraclass correlation coefficients for food consumption combined for three and pairwise two dietary assessment methods

	Three Methods	DHI vs. 24HR	DHI vs. WR	24HR vs. WR
Bread	0.49	0.43	0.52	0.56
Pastries	0.28	0.22	0.28	0.33
Vegetables, raw	0.28	0.18	0.31	0.48
Vegetables, cooked	0.18	0.08	0.24	0.23
Potatoes	0.38	0.32	0.47	0.32
Fruit	0.42	0.36	0.40	0.60
Nuts/seeds	0.36	0.32	0.39	0.38
Milk/dairy products	0.56	0.51	0.54	0.66
Eggs	0.38	0.32	0.51	0.27
Meat	0.32	0.30	0.34	0.30
Sausages/meat products	0.53	0.50	0.57	0.51
Fish/-products	0.35	0.33	0.43	0.26
Sweets	0.31	0.24	0.34	0.39
Ice cream	0.29	0.28	0.26	0.35
Water	0.57	0.53	0.53	0.67
Coffee/tea	0.65	0.66	0.62	0.69
Beer	0.71	0.67	0.74	0.72
Wine, sparkling wine	0.59	0.50	0.67	0.58

*DHI* diet history interviews, *24HR* 24-h recalls, *WR* weighed food record

### Comparison on the level of energy and nutrient intake

#### Median values

The diet history interviews showed higher estimates for median nutrient intakes in 14 out of 20 assessed nutrients compared to 24-h recalls and 12 out of 20 assessed nutrients compared to weighed food records. For energy intake, no differences between the dietary assessment methods could be shown as well as for intake of protein, fat, vitamin B12, and zinc (Fig. 2).

#### Differences

Pairwise differences of nutrient intake mirror results of food consumption. Again, the largest differences and highest effect sizes were found between diet history interviews and 24-h recalls as well as least differences and lowest effect sizes between 24-h recalls and weighed food records (Table 5). High relative differences in pairwise comparisons with diet history interviews for dietary fibre, retinol equivalents, folate equivalents, and vitamin C reflect the high consumption estimates of vegetables and fruit assessed with diet history interviews. Results for energy adjusted intake estimates (data not shown) did not deviate from the outcomes not adjusted for energy.

**Table 5:** Energy and nutrient intake and pairwise differences between the dietary assessment methods with effect size estimates

	DHI		24HR		WR		Difference of means 24HR-DHI				Difference of means DHI-WR				Difference of means 24HR-WR			
	Mean	Median	Mean	Median	Mean	Median	Mean	CI	%^a^	Effect size^d^	Mean	CI	%^b^	Effect size^d^	Mean	CI	%^c^	Effect size^d^
Energy (kcal/day)	2180.0	2086.6	2040.0	1969.8	2034.1	1998.9	− 139.9	− 190.9; − 93.1	− 6.4	0.2	146.2	99.1; 195.8	7.2	0.2	6.3	− 29.0; 42.2	0.3	0.0
Protein (g/day)	75.6	71.6	72.5	70.5	71.6	70.3	− 3.1	− 5.0; − 1.4	− 4.1	0.1	4.0	2.3; 5.8	5.6	0.2	0.9	− 0.5; 2.3	1.2	0.0
Fat (g/day)	81.8	77.0	78.1	75.1	80.5	77.2	− 3.7	− 6.3; − 1.3	− 4.5	0.1	1.4	− 0.8; 3.8	1.7	0.0	− 2.3	− 4.2; − 0.5	− 3.0	0.1
Carbohydrate (g/day)	261.8	246.5	235.7	229.1	232.2	225.4	− 26.1	− 32.3; − 20.4	− 10.0	0.3	29.7	23.8; 36.0	12.8	0.4	3.6	− 0.9; 8.0	1.5	0.1
Alcohol (g/day)	10.0	4.5	12.1	7.2	10.8	5.3	2.1	1.1; 3.0	21.1	0.2	− 0.8	− 1.6; 0.0	− 7.2	0.1	1.3	0.5; 2.2	11.0	0.1
Dietary fibre (g/day)	26.5	25.2	20.1	19.4	19.7	18.9	− 6.4	− 7.0; − 5.7	− 24.1	0.7	6.8	6.2; 7.4	34.3	0.8	0.4	0.0; 0.8	1.9	0.1
Cholesterol (mg/day)	295.2	275.6	282.6	263.3	291.5	278.9	− 12.5	− 23.4; − 3.1	− 4.2	0.1	3.8	− 5.8; 13.6	1.3	0.0	− 8.8	− 17.6; − 0.4	− 3.1	0.1
Retinol equiv. (mg/day)	1.9	1.6	1.2	1.1	1.4	1.1	− 0.7	− 0.8; − 0.6	− 35.6	0.6	0.5	0.4; 0.6	39.4	0.4	− 0.1	− 0.3; − 0.1	− 12.2	0.1
Vitamin E (mg/day)	13.1	11.5	10.4	9.9	11.2	10.3	− 2.7	− 3.3; − 2.2	− 20.8	0.4	1.9	1.5; 2.5	17.4	0.3	− 0.8	− 1.2; − 0.4	− 7.6	0.2
Thiamine (mg/day)	1.6	1.3	1.2	1.2	1.3	1.2	− 0.3	− 0.4; − 0.3	− 20.6	0.4	0.3	0.2; 0.3	20.9	0.3	− 0.1	− 0.1; 0.0	− 4.1	0.1
Riboflavin (mg/day)	1.8	1.6	1.4	1.3	1.4	1.3	− 0.4	− 0.4; − 0.4	− 21.1	0.5	0.3	0.2; 0.4	20.8	0.4	− 0.1	− 0.1; 0.0	− 5.1	0.1
Pyridoxine (mg/day)	2.0	1.7	1.5	1.4	1.6	1.5	− 0.5	− 0.6; − 0.4	− 24.1	0.5	0.4	0.3; 0.5	25.0	0.4	− 0.1	− 0.1; 0.0	− 5.4	0.1
Folate equiv. (µg/day)	304.1	269.8	208.3	202.2	237.1	216.8	− 95.8	− 108.2; − 85.2	− 31.5	0.6	67.0	55.7; 80.0	28.2	0.4	− 28.8	− 35.7; − 22.2	− 13.8	0.3
Vitamin B12 (µg/day)	5.0	4.5	4.8	4.5	4.8	4.3	− 0.2	− 0.4; 0.0	− 3.6	0.1	0.2	0.1; 0.4	3.9	0.1	0.0	− 0.2; 0.2	0.2	0.0
Vitamin C (mg/day)	165.1	143.6	109.7	100.0	118.1	103.0	− 55.4	− 62.3; − 48.5	− 33.5	0.6	47.0	39.3; 54.1	39.8	0.5	− 8.4	− 15.4; − 3.3	− 7.7	0.1
Potassium (mg/day)	3541.9	3439.8	2856.7	2783.7	2895.0	2843.4	− 685.2	− 759.6; − 614.2	− 19.3	0.7	647.2	577.3; 722.0	22.3	0.7	− 38.0	− 89.1; 14.4	− 1.3	0.1
Calcium (mg/day)	1015.9	932.9	830.2	798.7	843.7	803.1	− 185.7	− 213.7; − 159.4	− 18.3	0.5	172.4	145.8; 200.7	20.4	0.5	− 13.4	− 34.4; 7.8	− 1.6	0.0
Magnesium (mg/day)	390.6	375.4	329.4	322.9	330.9	321.7	− 61.2	− 69.4; − 53.3	− 15.7	0.6	59.7	52.0; 67.8	18.0	0.6	− 1.5	− 8.0; 5.1	− 0.5	0.0
Iron (mg/day)	12.4	11.8	11.3	10.9	11.4	11.3	− 1.1	− 1.4; − 0.8	− 8.7	0.3	1.0	0.7; 1.3	8.8	0.3	− 0.1	− 0.3; 0.2	− 0.6	0.0
Zinc (mg/day)	10.9	10.3	10.0	9.7	10.1	9.8	− 0.9	− 1.2; − 0.7	− 8.5	0.3	0.9	0.6; 1.1	8.5	0.2	− 0.1	− 0.3; 0.1	− 0.8	0.0

*DHI* diet history interviews, *24HR* 24-h recalls, *WR* weighed food record, *CI* confidence intervals

^a^DHI = 100%

^b^WR = 100%

^c^24HR = 100%

^d^Cohen’s *d*

#### Agreement: ICC

Strong agreement between all three dietary assessment methods was reached for alcohol intake, followed by a moderate agreement for intake of energy, macronutrients, dietary fibre, cholesterol, and minerals (Table 6). Only fair agreement was found for the vitamins. Retinol equivalents showed the least agreement with a correlation coefficient of 0.19. The paired analysis showed the strongest correlation coefficients between 24-h recalls and weighed food records in comparison with both other combinations. Again, this is in accordance with food consumption results.

**Table 6:** Intraclass correlation coefficients for energy and nutrient intake combined for three and pairwise two dietary assessment methods

	Three Methods	DHI vs. 24HR	DHI vs. WR	24HR vs. WR
Energy	0.56	0.52	0.53	0.66
Protein	0.54	0.50	0.55	0.60
Fat	0.52	0.46	0.53	0.60
Carbohydrate	0.52	0.49	0.48	0.63
Alcohol	0.65	0.58	0.72	0.65
Dietary fibre	0.46	0.38	0.44	0.66
Cholesterol	0.48	0.45	0.49	0.50
Retinol equivalents	0.19	0.15	0.23	0.17
Vitamin E	0.31	0.20	0.37	0.40
Thiamine	0.25	0.20	0.25	0.40
Riboflavin	0.38	0.31	0.37	0.50
Pyridoxine	0.29	0.22	0.28	0.49
Folate equivalents	0.26	0.18	0.25	0.46
Vitamin B12	0.36	0.36	0.37	0.35
Vitamin C	0.29	0.23	0.30	0.36
Potassium	0.47	0.39	0.44	0.65
Calcium	0.45	0.39	0.47	0.52
Magnesium	0.50	0.42	0.50	0.60
Iron	0.48	0.42	0.49	0.54
Zinc	0.49	0.43	0.51	0.54

*DHI* diet history interviews, *24HR* 24-h recalls, *WR* weighed food record

### Comparison of under- and overreporting

The proportion of underreporting is 23% for the diet history interviews, 22% for the weighed food records, and 16% for the 24-h recalls. 7% of participants underreported in each of the three methods, while 10% exclusively underreported in the diet history interviews, 7% in the weighed food records, and 4% in the 24-h recalls. For overreporting, the proportions are 4% (*n* = 27) for the diet history interviews and < 1% for the weighed food records (*n* = 4) and 24-h recalls (*n* = 3). The overreporting subgroups were not further evaluated because of the small sample sizes.

#### Food consumption

For each dietary assessment method relative differences between plausible reporters and underreporters were calculated for each food group to evaluate whether special food groups are more affected by underreporting than others (selective underreporting) (Table 7). Differences exceeding 25% were found for the food groups bread, pastries, nuts/seeds, milk/dairy products, sausages/meat products, sweets, ice cream, and alcoholic beverages in all dietary assessment methods. Weighed food records and diet history interviews depicted for most food groups higher amounts of relative differences between plausible reporters and underreporters compared to 24-h recalls.

**Table 7:** Food consumption of underreporters and plausible reporters and relative differences between these for each dietary assessment method

	Diet history interviews					24-h recalls					Weighed food records				
	Underreporters *n* = 158		Plausible reporters *n* = 519			Underreporters *n* = 105		Plausible reporters *n* = 572			Underreporters *n* = 150		Plausible reporters *n* = 527		
	Mean	CI	Mean	CI	Diff.^a^	Mean	CI	Mean	CI	Diff.^a^	Mean	CI	Mean	CI	Diff.^a^
	(g/day)	(g/day)	(g/day)	(g/day)	(%)	(g/day)	(g/day)	(g/day)	(g/day)	(%)	(g/day)	(g/day)	(g/day)	(g/day)	(%)
Bread	110	103; 118	169^b^	162; 178	− 35	95	88; 103	141^b^	136; 145	− 32	102	96; 110	138^b^	133; 144	− 26
Pastries	20	17; 24	42^b^	39; 46	− 53	45	40; 50	70^b^	67; 73	− 36	43	37; 49	73^b^	69; 78	− 41
Vegetables, raw	134	119; 151	147	138; 157	− 9	55	47; 66	71^b^	67; 75	− 22	55	47; 65	81^b^	76; 87	− 32
Vegetables, cooked	93	84; 104	114^b^	107; 121	− 18	66	62; 72	70	68; 72	− 5	57	51; 65	80^b^	76; 84	− 28
Potatoes	61	55; 67	83^b^	79; 87	− 27	57	51; 64	67^b^	64; 70	− 15	63	55; 72	80^b^	76; 85	− 22
Fruit	211	186; 244	269^b^	253; 287	− 21	131	113; 151	170^b^	160; 180	− 23	110	94; 129	157^b^	147; 169	− 30
Nuts/seeds	1	1; 2	5^b^	4; 7	− 77	1	1; 2	4^b^	3; 4	− 64	1	1; 2	4^b^	3; 5	− 70
Milk/dairy products	128	110; 149	230^b^	211; 252	− 45	112	93; 135	180^b^	169; 193	− 38	108	92; 128	175^b^	162; 189	− 38
Eggs	11	9; 13	14^b^	13; 16	− 24	9	7; 11	12^b^	11; 13	− 24	11	9; 13	14^b^	13; 16	− 26
Meat	31	27; 35	45^b^	42; 49	− 32	44	40; 49	51	49; 53	− 13	35	31; 40	46^b^	43; 49	− 23
Sausages/meat products	35	31; 40	60^b^	56; 65	− 42	47	41; 53	65^b^	62; 68	− 28	48	42; 54	69^b^	65; 73	− 31
Fish/-products	17	15; 20	26^b^	24; 28	− 34	16	14; 19	22^b^	21; 23	− 24	15	12; 19	19	18; 22	− 22
Sweets	11	9; 15	21^b^	19; 24	− 47	8	6; 10	14^b^	13; 15	− 45	7	6; 10	14^b^	13; 16	− 49
Ice cream	4	3; 6	9^b^	8; 11	− 54	4	2; 7	8^b^	6; 9	− 50	4	3; 6	8^b^	7; 10	− 52
Water	1309	1190; 1434	1201	1133; 1273	9	1222	1085; 1370	963^b^	913; 1015	27	1131	1018; 1251	921^b^	862; 984	23
Coffee/tea	427	368; 494	535^b^	496; 578	− 20	406	349; 474	536^b^	506; 565	− 24	314	276; 357	430^b^	403; 458	− 27
Beer	68	48; 105	143^b^	121; 171	− 53	111	81; 155	172	152; 194	− 35	58	39; 84	161^b^	138; 189	− 64
Wine, sparkling wine	39	27; 61	60	50; 76	− 35	38	28; 52	64^b^	57; 72	− 41	23	16; 33	67^b^	58; 80	− 66

*CI* confidence interval calculated with bootstrap method

^a^Diff. = relative difference between plausible reporters and underreporters (underreporters = 100%)

^b^Mann–Whitney *U* test between underreporters and plausible reporters *p* < 0.05

#### Energy and nutrient intake

For underreporting of energy and nutrient intake, relative differences between plausible reporters and underreporters were mostly 30–40% in each dietary assessment method (Table 8). Again, weighed food records and diet history interviews reached higher relative differences for most food groups compared to 24-h recalls. Alcohol intake was found with the highest deviations between plausible reporters and underreporters (82% for the weighed food records, 52% for the diet history interviews, and 38% for the 24-h recalls).

**Table 8:** Energy and nutrient intake of underreporters and plausible reporters and relative differences between these for each dietary assessment method

	Diet history interviews					24-h recalls					Weighed food records				
	Underreporters *n* = 158		Plausible reporters^b^ *n* = 519			Underreporters *n* = 105		Plausible reporters^b^ *n* = 572			Underreporters *n* = 150		Plausible reporters^b^ *n* = 527		
	Median	CI	Median	CI	%Diff^a^	Median	CI	Median	CI	%Diff^a^	Median	CI	Median	CI	%Diff^a^
Energy (kcal/day)	1421	1371; 1481	2294	2211; 2345	38	1360	1230; 1404	2080	2026; 2145	35	1353	1304; 1416	2157	2100; 2197	37
Proteine (g/day)	49.6	47.6; 52.3	78.3	76.3; 81.0	37	50.3	46.8; 54.8	73.7	71.7; 75.3	32	52.4	50.1; 54.8	74.8	73.2; 77.1	30
Fat (g/day)	50.9	47.2; 54.2	83.9	81.5; 87.1	39	46.6	44.7; 49.6	79.5	77.6; 82.8	41	51.8	48.8; 54.6	83.3	80.8; 86.1	38
Carbohydrate (g/day)	171	160; 184	272	265; 279	37	159	151; 172	241	234; 246	34	156	152; 162	244	237; 251	36
Alcohol (g/day)	2.6	1.7; 3.6	5.4	4.4; 6.5	52	4.8	3.2; 7.1	7.7	6.6; 8.8	38	1.4	0.3; 2.8	7.8	6.3; 8.8	82
Dietary fibre (g/day)	18.5	17.6; 20.3	27.1	26.3; 28.0	32	14.4	13.7; 15.1	20.4	19.7; 21.0	29	13.5	12.6; 14.7	20.1	19.4; 20.6	33
Cholesterol (mg/day)	179	172; 200	304	291; 316	41	187	170; 195	283	270; 293	34	192	174; 216	298	289; 307	36
Retinol equiv. (mg/day)	1.3	1.2; 1.4	1.8	1.7; 1.9	27	0.9	0.8; 0.9	1.2	1.1; 1.2	26	0.7	0.6; 0.8	1.2	1.2; 1.3	45
Vitamin E (mg/day)	8.2	7.4; 8.5	12.8	12.3; 13.8	36	7.2	6.1; 7.8	10.4	10.1; 10.8	31	7.2	6.6; 7.5	11.2	10.8; 11.7	36
Thiamine (mg/day)	0.9	0.9; 1.0	1.4	1.4; 1.5	35	0.9	0.8; 0.9	1.2	1.2; 1.3	27	0.9	0.8; 0.9	1.3	1.2; 1.3	31
Riboflavine (mg/day)	1.1	1.0; 1.1	1.7	1.7; 1.8	37	0.9	0.9; 1.0	1.4	1.4; 1.4	33	0.9	0.9; 1.0	1.4	1.4; 1.5	35
Pyridoxine (mg/day)	1.2	1.2; 1.3	1.9	1.8; 1.9	34	1.1	1.0; 1.1	1.5	1.4; 1.5	28	1.0	1.0; 1.1	1.6	1.5; 1.6	36
Folate equiv. (µg/day)	207	191; 217	293	280; 307	29	141	132; 150	211	207; 217	33	142	134; 156	234	228; 242	39
Vitamin B12 (µg/day)	2.9	2.7; 3.1	5.1	4.9; 5.3	43	3.4	3.0; 3.6	4.7	4.5; 4.9	27	3.2	2.9; 3.4	4.6	4.5; 4.8	31
Vitamin C (mg/day)	115	104; 124	154	144; 163	26	69.3	64; 79	105	100; 110	34	69.9	61; 81	112	106; 118	38
Potassium (mg/day)	2525	2432; 2737	3683	3554; 3800	31	1998	1871; 2220	2902	2822; 2970	31	1942	1877; 2027	3089	3004; 3160	37
Calcium (mg/day)	702	680; 746	1019	985; 1052	31	619	577; 656	833	812; 851	26	582	553; 644	865	834; 884	33
Magnesium (mg/day)	282	272; 295	405	396; 415	31	247	230; 258	337	327; 344	27	233	224; 248	344	336; 351	32
Iron (mg/day)	8.8	8.2; 9.2	12.8	12.4; 13.2	31	7.8	7.4; 8.3	11.4	11.1; 11.7	32	7.6	7.3; 7.9	12.0	11.7; 12.2	36
Zinc (mg/day)	7.4	7.1; 7.7	11.3	11.1; 11.7	35	7.0	6.6; 7.4	10.1	9.8; 10.3	30	7.1	6.7; 7.4	10.4	10.1; 10.6	32

*CI* confidence interval calculated with bootstrap method

^a^Diff. = relative difference between plausible reporters and underreporters (underreporters = 100%)

^b^Mann–Whitney *U* test between underreporters and plausible reporters p < 0.05

## Discussion

### Comparison on the level of food consumption

Estimated mean food consumption showed the largest differences between diet history interviews and 24-h recalls and least differences between 24-h recalls and weighed food records. In 7 of 18 food groups, diet history interviews showed higher consumption means than 24-h recalls and weighed food records.

Few other published studies describe food consumption of a diet history method in comparison with food records or 24-h recalls. Chinnock [27] validated a diet history questionnaire using a weighed food record as reference method in a group of 60 adults in Costa Rica. Mean food consumption assessed with the diet history questionnaire gave higher estimates for three of the 18 food groups compared with the weighed food records in men and for one food group in women. Sjöberg and Hulthen [28] compared results of a diet history questionnaire with an estimated 7-day food record from 51 girls 15–16 years of age. For most food groups (14 out of 20) they showed higher consumption assessed with the diet history questionnaire in comparison with food records. The number of in-between meals was higher using the diet history questionnaire. The authors assume that this contributes to the higher results of the diet history questionnaire regarding bread, fruit, and milk/dairy products. Van Liere et al. [29] compared a diet history questionnaire with the average of 9–12 single 24-h recalls carried out over one year in a group of 115 adult women. In 11 out of 18 food groups, the food consumption assessed with the diet history questionnaire was higher compared to the 24-h recalls.

A comparison of weighed food records and 24-h recalls was carried out by Bingham et al. [30]. Two types of 24-h recalls were compared with weighed food records in 160 women 50–65 years of age: a simple 24-h recall consisting of a blank sheet of paper and a structured 24-h recall with portion size assessments using photographs. In both types of 24-h recalls, higher consumption means for beverages were found in comparison with weighed food records. In the present study, this can be confirmed only for coffee/tea but not for water or alcoholic beverages.

As in the present study, Chinnock [27] found the best agreement coefficients between different methods for beverages. Possible reasons for the good agreement of beverages are the small variance of household measures, such as glasses, cups or bottles, and constant day-to-day habits of beverage consumption. In the present study, a significantly lower mean consumption for coffee/tea was found in weighed food records in comparison with both other methods. Possibly, a part of the participants noted the amount of the used coffee/tea powder instead of the consumed beverage. However, it is supposed that this fact contributes only to a minor degree to the lower mean coffee/tea consumption of weighed food records. For alcoholic beverages many non-consumers are usually observed. This fact is discussed as an explanation for strong correlation coefficients between different dietary assessment methods [12, 30]. In the present study, significant differences between beer consumption assessed with 24-h recalls and both other methods were found (Table 2). In addition, the highest alcohol intake was assessed with 24-h recalls and the lowest with diet history interviews (Table 5). This is in accordance with Stockwell et al. [31] who pointed out that recall methods which ask for the actual alcohol consumption usually show higher results than methods which require people to estimate their typical alcohol consumption over a longer time span. The authors argue that recall methods for actual consumption reduce the opportunity for memory loss and do not require complex judgments about the usual food consumption [31]. Furthermore, people tend to exclude high-intake occasions from consideration when they are asked to report their average alcohol consumption of a longer time span. Therefore, they rather report the lower “median” instead of the higher “mean” quantities [31, 32].

Data on food consumption may be biased by the tendency of individuals to overestimate foods rated as socially desirable and to underestimate foods rated as undesirable. Socially desirable answers lead to incorrect mean consumption estimates due to a systematic between-person error [2]. Underlying reasons for socially desirable answers are, e.g., attitudes towards foods, health, and gender aspects [33]. The tendency to overestimate foods perceived as socially desirable may be stronger when long-term dietary habits are assessed (by diet history interviews or food frequency questionnaires) instead of the actual consumption of single days (by 24-h recalls). Results of 173 women in the Nurses’ Health Study showed that a food frequency questionnaire tended to overestimate socially desirable foods in comparison with food records [34]. In the present study, these aspects may contribute to an overestimation of fruit and vegetables assessed by diet history interviews. Furthermore, the tendency to underestimate foods perceived as socially undesirable may contribute to lower estimates of pastries (e.g., cakes, cookies, pies, and spicy snacks). This is in accordance with other studies: van Liere et al. [29] revealed a lower consumption of cakes with diet history questionnaires in comparison with 24-h recalls and Sjöberg-Hulthen et al. [28] found a lower consumption of sweet baked goods with the diet history questionnaire than with food records.

In general, the results show that inhomogeneous food groups are more difficult to assess and show lower accordance between different dietary assessment methods. Difficulties in estimating quantities and frequencies arise particularly for inhomogeneous food groups, such as vegetables or pastries. If these difficulties occur, social desirability as mentioned above seems to have an important impact.

As expected, the diet history interviews in the present study covering weeks show the lowest numbers of non-consumption. The highest proportions of non-consumption are seen for weighed food records covering 8 days. In this regard, it should be emphasised that for 24-h recalls the distribution of usual food intake was calculated, leading to lower percentages of zero values as in the original data. Without calculating the usual intake, the 24-h recalls show the highest percentages of zero values. Rarely and/or seasonally consumed food groups, such as nuts/seeds, sweets, ice cream, or fish/-products are difficult to assess and show in the present study lower accordance between different methods than food groups consumed daily in more constant amounts, such as potatoes or coffee/tea.

In the present study, weighed food records and 24-h recalls show a better agreement and lower differences than pairwise comparisons with diet history interviews. Although mean values of 24-h recalls and weighed food records are comparable for most food groups, there are some well-known weaknesses of food records. Most prominent is the recording process itself, which can lead to changes of the usual eating pattern. In addition, food consumed away from home may be reported less detailed [2]. Workload is immense not only for participants but also for scientific staff, and because of rising costs, weighed food records are often not applicable for large study populations, especially as paper–pencil version. Web-based versions can reduce the workload for the scientific staff but not for participants. New devices such as mobile phones with integrated cameras or other technology assisted dietary assessment may lower the burden of record keeping in the future. Until now, 24-h recalls are more practicable for large study populations than weighed food records because of the participant burden.

The results of the diet history interviews, especially regarding fruit and vegetable consumption, are higher compared to 24-h recalls and weighed food records. The question arises which dietary assessment method is closest to the true food consumption. For hints regarding the validity of the estimated values, they were compared to data of food balance sheets of the production years 2005/2006 [35]. Production data do not account for any losses, e.g., waste or inedible parts; therefore, lower values of consumption data in comparison with production data are to be expected. However, the consumption data of diet history interviews are only slightly lower than production data. Therefore, this comparison suggests that the results of the diet history interviews regarding fruit and vegetable consumption are probably overestimated.

### Comparison on the level of energy and nutrient intake

In accordance with results of food consumption, the largest differences in nutrient intake were found between diet history interviews and 24-h recalls, least differences between 24-h recalls and weighed food records. The high relative differences in pairwise comparisons of 24-h recalls and weighed food records with diet history interviews for dietary fibre, retinol equivalents, folate equivalents, and vitamin C reflect the high consumption estimates of vegetables and fruit assessed with diet history interviews. The higher carbohydrate intake assessed with diet history interviews in comparison with 24-h recalls and weighed food records can be explained by a higher consumption estimate of fruit juices (results not shown) beside the higher consumption estimates of bread and fruit.

Several studies report higher intakes of energy and nutrients assessed by diet history interviews in comparison with food records [27, 28, 36–40] or 24-h recalls [29]. The relative differences described in these studies are comparable to the presented results. Few studies observed similar or lower energy and nutrient intakes with dietary history interviews in comparison with food records [41–43] or 24-h recalls [12]. Regarding 24-h recalls and food records, several studies found comparable or only slightly different results between these two methods [30, 44–46]. This is confirmed by the present study which also shows comparable nutrient intake estimates of 24-h recalls and weighed food records.

Several studies found low correlation coefficients between two methods for vitamin A [27, 29, 30, 39]. A possible reason for these low correlation coefficients is the large random variation in the daily intake of vitamin A [2] and the large inhomogeneity of the food groups fruit and vegetables.

### Comparison of underreporting

In the present study, underreporting was assessed by the ratio of energy intake and individually calculated resting metabolic rate. The proportion of underreporting was lowest in 24-h recalls with 16%, while diet history interviews and weighed food records showed a similar average rate of 23 and 22%, respectively.

In other studies, wide ranges for the proportion of underreporting can be found for every dietary assessment method: 32–51% for diet history interviews [12, 47], 12–44% for estimated food records [8, 48–50], 14–46% for weighed food records [12, 49, 51], and 7% up to more than 50% for 24-h recalls [8, 49, 52–54]. A review of 37 studies comparing misreporting in estimated and weighed food records and 24-h recalls concludes that the underestimation of energy intake is similar in all three assessment methods [49]. Another review also demonstrates that estimates of dietary intake assessed by food records, 24-h recalls, and diet history questionnaires are biased towards underreporting and that neither prospective nor retrospective methods are consistently better than the other in this regard [55].

To address the question whether underreporting is differently associated with specific food in one of the three dietary assessment methods, a comparison between underreporters and plausible reporters was made within each assessment method. In the literature, foods rich in sugar and/or fat as well as alcoholic beverages are often found to be underreported [56–58]. In the present study, the consumption of pastries, sweets, and ice cream is also to a considerable amount lower in underreporters than in plausible reporters in all three assessment methods. Furthermore, food groups with a high social desirability, such as vegetables and fruit are not expected to be underreported in considerable amounts [59–61]. Nevertheless, in weighed food records of the present study underreporters reported raw and cooked vegetables to a sizeable lower extent in comparison with plausible reporters. This could not be found for diet history interviews (especially raw vegetables) and 24-h recalls (especially cooked vegetables). Vegetables as a highly inhomogeneous food group were mostly consumed in mixed dishes and, therefore, complex to protocol. Presumably, two opposed categories of behaviour regarding underreporting become evident. First, the ‘healthy’ perception of these food groups resulting in a high social desirability and potentially over-recording is contrary to the inconvenience and time consumption of protocolling. Recording fatigue may, therefore, be a possible reason for underreporting vegetables in the weighed food records as well as a change in eating behaviour leading to undereating and so to reactivity bias [49, 59].

Overall, smaller differences between underreporters and plausible reporters were found for 24-h recalls, while weighed food records and diet history interviews exhibited higher percentages of differences between underreporters and plausible reporters. Therefore, the general expectation that the extent of underreporting would be the lowest with weighed food records (because estimation of portion sizes and frequencies is not required for this method) could not be confirmed.

Mean nutrient intake of underreporters and plausible reporters differed for most nutrients between 30–40% for all three methods giving no further insights regarding differences between the three methods. Altogether, no major differences between the three assessment methods regarding underreporting could be found, and underreporting is a problem in any method.

### Strengths and limitations

The sample of 677 participants 14–80 years of age completing all three dietary assessment methods has to be seen as strength. To enhance comparability, all procedures regarding data handling were standardized as much as possible. Equal estimation of serving sizes was supported by applying the identical picture book for diet history interviews and 24-h recalls. Regarding time frame, participants accomplished all three assessment methods on average within 45 days. However, the period of time under consideration is longer, because the diet history interview requests food consumption the 4 weeks before the interview. This adds up to the total average study period of about 2 and a half months for each participant. Therefore, seasonal influences cannot be excluded. Another limitation of this comparison of dietary assessment methods is that biomarkers could not be incorporated in the study. Biomarkers for food or nutrient intake have errors independent from that of dietary assessment methods and, therefore, would be a helpful addition for the interpretation of the results. A further limitation is seen in the order in which the three methods were applied. For organizational reasons, the three assessment methods could not be applied in a randomized order; therefore, a trainings effect is possible. To complete all three dietary assessment methods, subjects must be highly cooperative. In comparison with all participants of the German National Nutrition Survey II, subjects of the current analysis exhibit a higher education and the proportion of women is larger (Table 1). The selection of participants might have an influence on the results.

## Conclusions

The present study revealed that 24-h recalls and weighed food records showed smaller differences and better agreement for food consumption and nutrient intake than pairwise comparisons with diet history interviews. The strength of the diet history interview to assess the usual food consumption also imbeds its limitation: diet history interviews require complex judgments regarding consumed quantities over a long time period, whereas 24-h recalls only refer to the day before the interview, while weighed food records do not depend on a memory effort. In diet history interviews inhomogeneous food groups (e.g., vegetables) and mixed dishes impede estimation of quantities and frequencies, probably reinforcing the influence of social desirability. In weighed food records these foods may have caused recording fatigue, undereating, or underreporting. For dietary assessment methods encompassing a short time span such as 24-h recalls, the difficulty of assessing rarely eaten foods is a major limitation. This is outweighed by the low memory effort, which is probably diminishing the influence of social desirability, and the low respondent burden. In this regard, the present results support the recommendation of the European Food Safety Authority [6, 7] to apply multiple 24-h recalls for national nutrition surveys.

All this underlines that the choice of the adequate dietary assessment method depends on the research question and the foods and nutrients to be studied. New devices such as mobile phones with integrated cameras or other technology assisted dietary assessment methods may lower the burden for participants in the future. However, identifying and mitigating measurement error stays even then an important task.

## Appendix

See Table 9.

**Table 9:** Description of food groups

Food group	Description
Bread	Rolls, toast, rusk
Pastries	Cake, pies, tarts, cookies salty pastries such as filled puff paste, and snacks such as peanut flips, crackers, tortilla chips
Vegetables, raw	Incl. mushrooms and legumes (sprouts) incl. frozen vegetables
Vegetables, cooked	Incl. mushrooms and legumes incl. sauerkraut
Potatoes	Incl. chips, hash browns, mashed potatoes, potato crisps
Fruit	Incl. unsweetened frozen fruit
Nuts/seeds	Hazelnuts, peanuts, almonds, sesame etc. processed products such as peanut butter, salted, or roasted nuts
Milk/dairy products	Incl. cacao drinks, milkshakes, yoghurt, (sour) cream, kefir, whey without cheese and curd cheese
eggs	Incl. fried and cooked eggs, eggs in dishes without eggs in pastries, soups and sauces
meat	E.g., pork, beef, lamb, turkey or chicken
Sausages/meat products	Incl. meat sauces, meatballs, smoked pork chops, cured pork
Fish/-products	Incl. shellfish, caviar und canned fish
Sweets	E.g., chocolate, candy, jelly baby, bars, pralines, liquorice
Ice cream	Incl. soft serve ice cream, frozen yoghurts
Water	Mineral water, tap water
Coffee/tea	Incl. cappuccino black and green tea, but not herbal tea and fruit infusions
Beer	Incl. mixed beer drinks
Wine, sparkling wine	Without alcopops, cocktails and alcoholic beverages above Vol.15%

## Footnotes

1 The EPIC-SOFT program is now renamed to GloboDiet.
